# The relationship between retroperitoneal lymphadenectomy and survival in advanced ovarian cancer patients

**DOI:** 10.1186/s12885-020-07144-1

**Published:** 2020-07-13

**Authors:** Chenyan Fang, Yingli Zhang, Lingqin Zhao, Xi Chen, Liang Xia, Ping Zhang

**Affiliations:** 1 Department of Gynecological Oncology, Cancer Hospital of University of Chinese Academy of Sciences (Zhejiang Cancer Hospital), Institute of Cancer Research and Basic Medicine (IBMC), Chinese Academy of Sciences, 1 Banshan East Road, Hangzhou, 310022 Zhejiang Province China; 2 Department of Neurosurgery, Cancer Hospital of University of Chinese Academy of Sciences (Zhejiang Cancer Hospital), Institute of Cancer Research and Basic Medicine (IBMC), Chinese Academy of Sciences, 1 Banshan East Road, Hangzhou, 310022 Zhejiang Province China

**Keywords:** Advanced ovarian Cancer, Optimal Cytoreduction, Survival, Systematic retroperitoneal lymphadenectomy

## Abstract

**Background:**

Systematic retroperitoneal lymphadenectomy has been widely used in the surgical treatment of advanced ovarian cancer patients. Nevertheless, the corresponding therapeutic may not provide a survival benefit. The aim of this study was to assess the effect of systematic retroperitoneal lymphadenectomy in such patients.

**Methods:**

Patients with advanced ovarian cancer (stage III-IV, according to the classification presented by the International Federation of Gynecology and Obstetrics) who were admitted and treated in Zhejiang Cancer Hospital from January 2004 to December 2013 were enrolled and reviewed retrospectively. All patients were optimally or suboptimally debulked (absent or residual tumor < 1 cm) and divided into two groups. Group A (no-lymphadenectomy group, *n* = 170): patients did not undergo lymph node resection; lymph nodes resection or biopsy were selective. Group B (*n* = 240): patients underwent systematic retroperitoneal lymphadenectomy.

**Results:**

A total of 410 eligible patients were enrolled in the study. The patients’ median age was 51 years old (range, 28–72 years old). The 5-year overall survival (OS) and 2-year progression-free survival (PFS) rates were 78 and 24% in the no-lymphadenectomy group and 76 and 26% in the lymphadenectomy group (*P* = 0.385 and 0.214, respectively). Subsequently, there was no significant difference in 5-year OS and 2-year PFS between the two groups stratified to histological types (serous type or non-serous type), the clinical evaluation of negative lymph nodes or with macroscopic peritoneal metastasis beyond pelvic (IIIB-IV). Multivariate Cox regression analysis indicated that systematic retroperitoneal lymphadenectomy was not a significant factor influencing the patients’ survival. Patients in the lymphadenectomy group had a higher incidence of postoperative complications (incidence of infection treated with antibiotics was 21.7% vs. 12.9% [*P* = 0.027]; incidence of lymph cysts was 20.8% vs. 2.4% [*P* < 0.001]).

**Conclusions:**

Our study showed that systematic retroperitoneal lymphadenectomy did not significantly improve survival of advanced ovarian cancer patients with residual tumor < 1 cm or absent after cytoreductive surgery, and were associated with a higher incidence of postoperative complications.

## Background

Ovarian cancer is the second most common cancer in females worldwide, with about 225,500 new cases occurred globally every year, and its mortality rate is as high as 47%, which is higher than that of any other gynecological malignancies [1]. Ovarian cancer is often diagnosed at an advanced stage due to the lack of effective measures for early detection and its late symptomatology [2, 3]. To our knowledge, ovarian cancer spreads in two ways: intraabdominally (direct extension and exfoliation of the primary tumor in the peritoneal cavity) and retroperitoneally (through the lymphatic channels). Retroperitoneal lymphatic spread has been reported to be a common feature both in early and advanced ovarian cancer patients, the rate of lymph node metastasis is totally about 20–41%, which can reach up to 50–80% in advanced patients (FIGO stage III-IV) [4, 5]. Considering the optimal cytoreduction, comprehensive staging and the guidance of postoperative treatment, the guidelines published by the National Comprehensive Cancer Network (NCCN) recommend that systematic retroperitoneal lymphadenectomy (including pelvic and paraaortic lymphadenectomy) should be included in the primary surgery of early ovarian cancer patients. Nevertheless, studies on whether systematic retroperitoneal lymphadenectomy improve the prognosis of patients with advanced ovarian cancer provide conflicting results. Numerous retrospective studies have shown that retroperitoneal lymphadenectomy can improve prognosis in patients with advanced ovarian cancer [6–10], while some randomized controlled trials did not show survival benefit of systematic retroperitoneal lymphadenectomy in advanced ovarian cancer patients [11, 12].

In addition, retroperitoneal lymphadenectomy may increase intraoperative and postoperative complications, such as bleeding, vascular injury, lymphocysts, infection, intestinal fistula, chylous fistula, lower limb edema, pulmonary embolism, repeat laparotomy and post-operative mortality. Hence, the role of retroperitoneal lymphadenectomy in advanced ovarian cancer surgery deserves our attention.

In view of the results above, we performed a retrospective analysis of this issue again.

## Methods

All primary ovarian cancer patients treated in Zhejiang Cancer Hospital from January 2004 to December 2013 were retrospectively reviewed and a total of 410 patients with International Federation of Gynecology and Obstetrics (FIGO, 2014) stages III and IV were enrolled in this study. All of them underwent complete surgical staging including total abdominal hysterectomy, bilateral salpingo-oophorectomy, and omentectomy, additionally, to achieve optimal debulking, surgical procedures like retroperitoneal lymph node resection (systematic retroperitoneal lymphadenectomy, selective lymph node resection or biopsy), resection of other organs (e.g., sigmoid colon, rectum, small intestine, liver, spleen, diaphragm, urinary tracts) were performed. Furthermore, to eliminate the effect of large-volume residual disease on patients’ survival, all patients included in this analysis were optimally debulked (no gross residual disease) or sub optimally debulked (residual disease < 1 cm). Patients who underwent initial surgical exploration elsewhere or received neoadjuvant chemotherapy before surgery were excluded.

Patients in our analysis were divided into two groups. Group A (*n* = 170) (no-lymphadenectomy group): patients did not undergo lymph node resection; or lymph nodes resection or biopsy were selective. Group B (*n* = 240): patients underwent systematic retroperitoneal lymphadenectomy. And patients were divided into two histological types, serous and non-serous. Lymph nodes were diagnosed by intraoperative palpation and preoperative imaging (computed tomography scan, positron emission tomography-computed tomography, magnetic resonance imaging and ultrasound).

This study was approved by the Medical Ethics Committee of Zhejiang Cancer Hospital. No written informed consent was obtained from the patients due to the retrospective nature of the study. Data were retrospectively retrieved from hospital records, telephone interview or out-patient interview, including age, the level of serum cancer antigen 125 (CA-125), FIGO stage, surgical information (e.g., diameter of residual tumor, details of lymphadenectomy, intraoperative blood loss), histological subtype, intraoperative and postoperative complications, primary systemic therapy, and follow-up information.

Progression-free survival (PFS, the time from primary surgery to the date of first recurrence, date of death or date of last contact) and overall survival (OS, the time from primary surgery to the date of death, or date of last contact) were used to assess the patients’ survival.

### Statistical analysis

In the present study, OS, PFS, and the incidence of intraoperative and postoperative complications were selected as primary outcomes. All statistical analyses were carried out using the Statistical Package for Social Sciences (SPSS) statistical software (version 17.0). Categorical data were assessed using chi-square test or Fisher’s exact test. Multivariate Cox regression model were used to evaluate the influences of different covariates on OS and PFS, and were expressed as hazard ratio (HR). Meanwhile, survival curves were assessed using the Kaplan-Meier method, and the difference in survival was evaluated using the log-rank test. A two-sided *P* < 0.05 was considered statistically significant.

## Results

### Patient characteristics

A total of 410 advanced ovarian tumor patients were analyzed in this study, 170 cases in Group A and 240 in Group B, and the characteristics of the two groups are listed in Table 1.

**Table 1:** Patient characteristics

	No	GroupA	GroupB	*P*-value
Total	410	170	240	
Age (years)				0.257
Median		54	51	
Range		29–72	28–71	
Serum CA-125 level (U/mL)				0.532
Median		606.8	455.1	
Range		13–6743	6–10,000	
Intraoperative blood loss (ml)				0.889
Mean		537.7 ± 335.3	542.5 ± 352.4	
Range		100–1500	100–2200	
Histology				0.475
Serous	320	136 (80%)	184 (76.7%)	
Clear cell	6	4 (2.4%)	2 (0.8%)	
Endometrioid	46	16 (9.4%)	30 (12.5%)	
Mucinous	24	10 (5.9%)	14 (5.8%)	
Others	14	4 (2.4%)	10 (4.2%)	
FIGO stage				0.686
III	342	140 (82.4%)	202 (84.2%)	
IV	68	30 (17.6%)	38 (15.8%)	

The median age of patients in Groups A and B was 54 (29–72) and 51 (28–71) years old, respectively. The median serum CA-125 level was 606.8 U/mL (13–6743 U/ mL) in Group A and 455.1 U/mL (6–10,000 U/mL) in Group B. The majority of patients in Groups A and B were at FIGO stage III (82.4% of Group A and 84.2% of Group B), and a few cases were at stage IV (17.6% of Group A and 15.8% of Group B). Out of 410 total patients, serous tumors were the most common pathological subtype (*n* = 320; 78%), followed by endometrioid (*n* = 46; 11.2%), mucinous (*n* = 24; 5.9%), clear cell (*n* = 6; 1.5%) and 14 patients (3.4%) had others histological types. In addition, the mean intraoperative blood loss in Group B was slightly higher than that in Group A (542.5 ± 352.4 vs 537.7 ± 335.3 ml). There was no significant difference in the patients’ clinical characteristics between the two groups, including the age (*P* = 0.257), median serum CA-125 level (*P* = 0.532), intraoperative blood loss (*P* = 0.889), FIGO stage (*P* = 0.686), or pathological type (*P* = 0.475).

The postoperative complications and primary systemic treatment in Groups A and B are summarized in Table 2.

**Table 2:** Postoperative Complications and Primary Systemic Treatment

Complication or Treatment	Group A	Group B	*P*-value
	(*N* = 170)	(*N* = 240)	
Complication			
Infection treated with antibiotics	22 (12.9%)	52 (21.7%)	0.027
Thrombosis	4 (2.4%)	6 (2.5%)	0.924
Lymph cysts	4 (2.4%)	50 (20.8%)	< 0.001
Intestinal fistula	2 (1.2%)	4 (1.7%)	0.684
Repeat laparotomy for complications	2 (1.2%)	6 (2.5%)	0.340
Postoperative bleeding	0 (0)	2 (0.8%)	0.233
Primary systemic therapy			0.100
Paclitaxel and platinum	98 (57.6%)	160 (66.7%)	
Docetaxel and platinum	68 (40%)	76 (31.7%)	
Other systemic treatment	2 (1.2%)	4 (1.7%)	
No systemic treatment	2 (1.2%)	0 (0)	

It was found that the patients in lymphadenectomy group had a higher incidence of postoperative complications than those in no-lymphadenectomy group. Especially for the incidence of infection treated with antibiotics (21.7% [52 of 240 patients] vs. 12.9% [22 of 170 patients], *P* = 0.027) and the incidence of lymph cysts (20.8% [50 of 240] vs. 2.4% [4 of 170], *P* < 0.001). In addition, the main reason for repeat laparotomy of complications in Group B (2.5% [6 of 240 patients]) was postoperative bleeding, intestinal fistula or lymph cysts, and the main reason in Group A (1.2% [2 of 170 patients]) was fistula.

With respect to primary systemic treatment after cytoreductive surgery, the majority of patients received adjuvant chemotherapy, 98.4% of the patients in Group B and 97.6% of those in Group A were treated with paclitaxel or docetaxel and platinum. No significant difference was found between the two groups (*P* = 0.100) as well.

### Survival

**Fig. 1:**
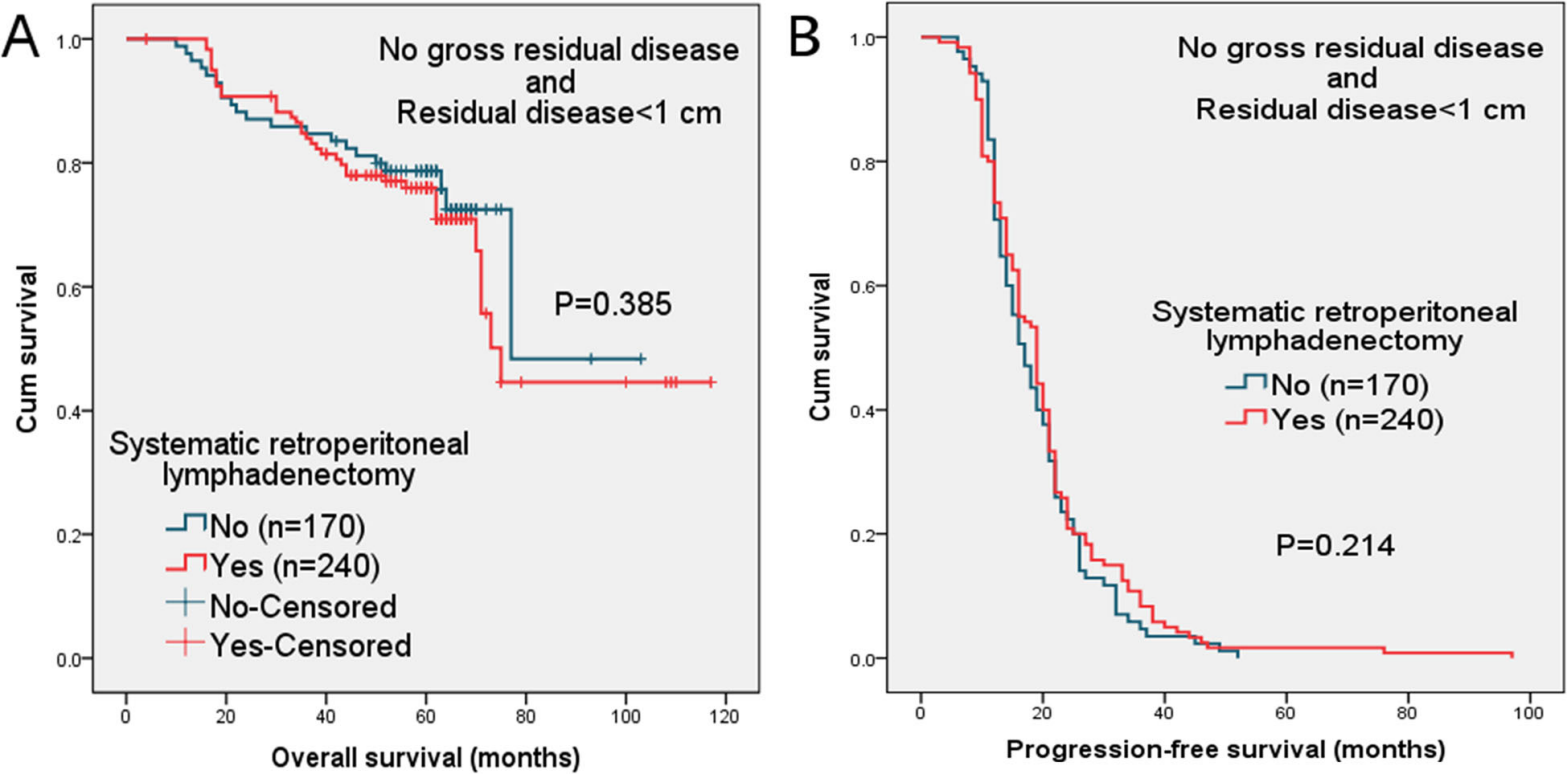
(**a**) Overall survival (OS) and (**b**) progression-free survival (PFS) in patients with or without systematic retroperitoneal lymphadenectomy, confining analysis to patients with no gross residual disease and residual disease < 1 cm

The 5-year OS and 2-year PFS rates were 78 and 24% in no-lymphadenectomy group and 76 and 26% in lymphadenectomy group (*P* = 0.385 and 0.214, respectively). The survival curves of these two groups were examined by Kaplan–Meier analysis, as shown in Fig. 1.

#### Without residual tumor

**Fig. 2:**
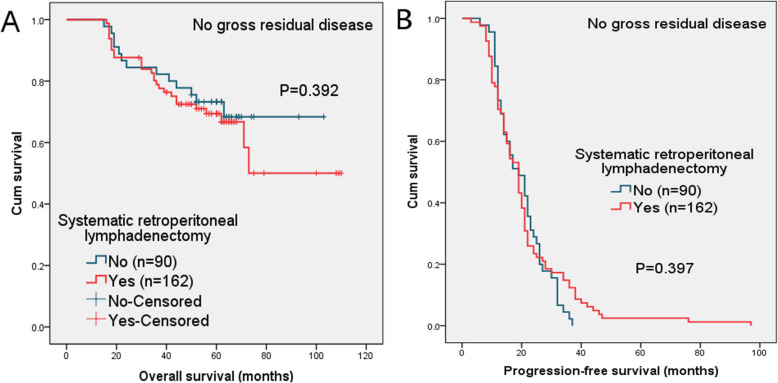
(**a**) Overall survival (OS) and (**b**) progression-free survival (PFS) in patients with no gross residual disease

When patients without residual tumor were analyzed, the 5-year OS and 2-year PFS rates were 73 and 31% in no-lymphadenectomy group and 69 and 26% in lymphadenectomy group, the difference was not statistically significant (*P* = 0.392 and 0.397, respectively). The survival curves are displayed in Fig. 2.

#### Histological type (serous or non-serous type)

**Fig. 3:**
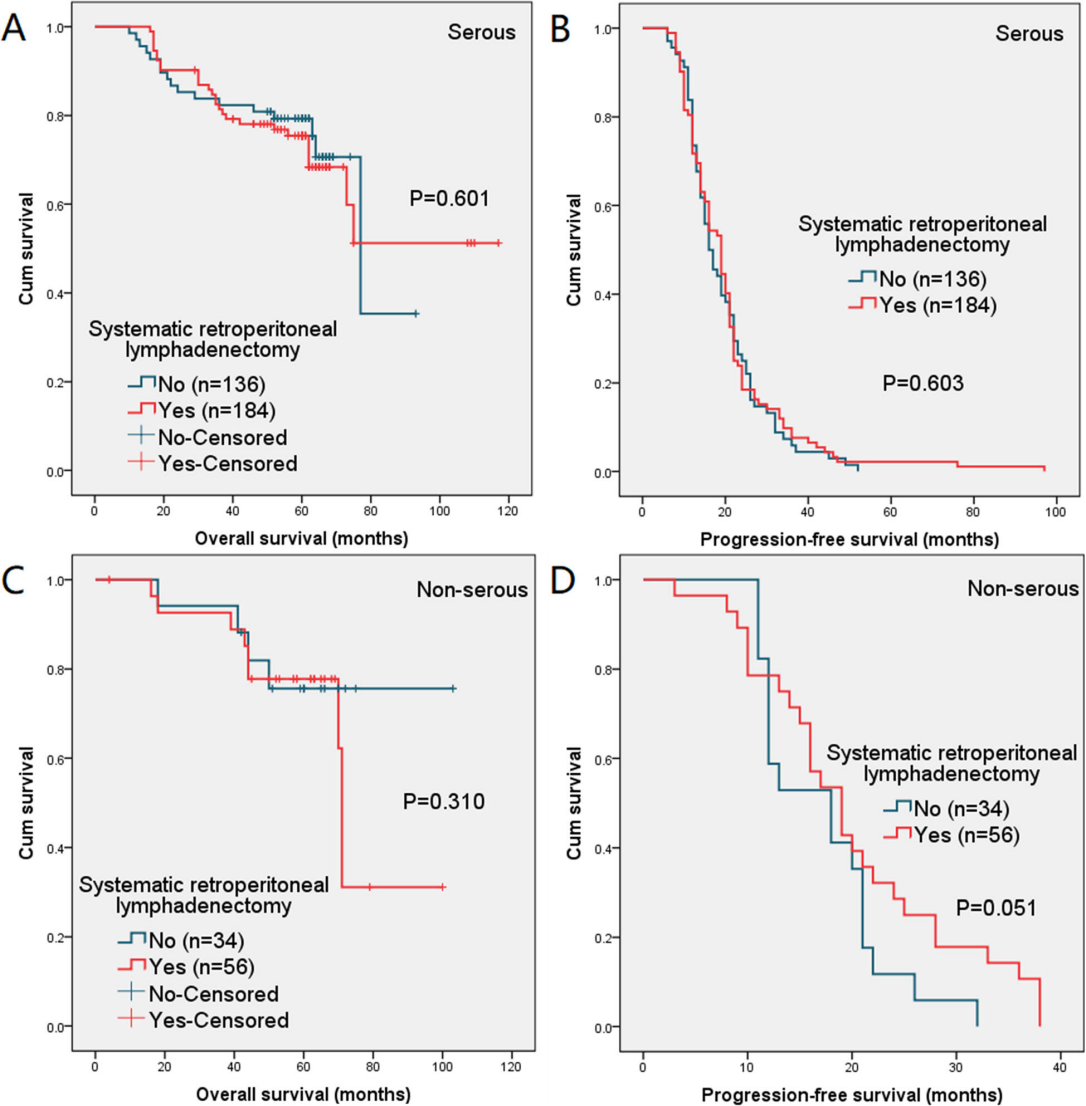
(**a**) Overall survival (OS) and (**b**) progression-free survival (PFS) in patients with serous type and (**c**) overall survival (OS) and (**d**) progression-free survival (PFS) in patients with non-serous type

Similarly, when confining analysis to patients with serous type or non-serous type, the difference in 5-year OS and 2-year PFS between the two groups was no significant (serous: *P* = 0.601 and 0.603, non-serous: *P* = 0.310 and 0.051). The survival curves are illustrated in Fig. 3.

#### Clinical evaluation for lymph nodes (negative)

**Fig. 4:**
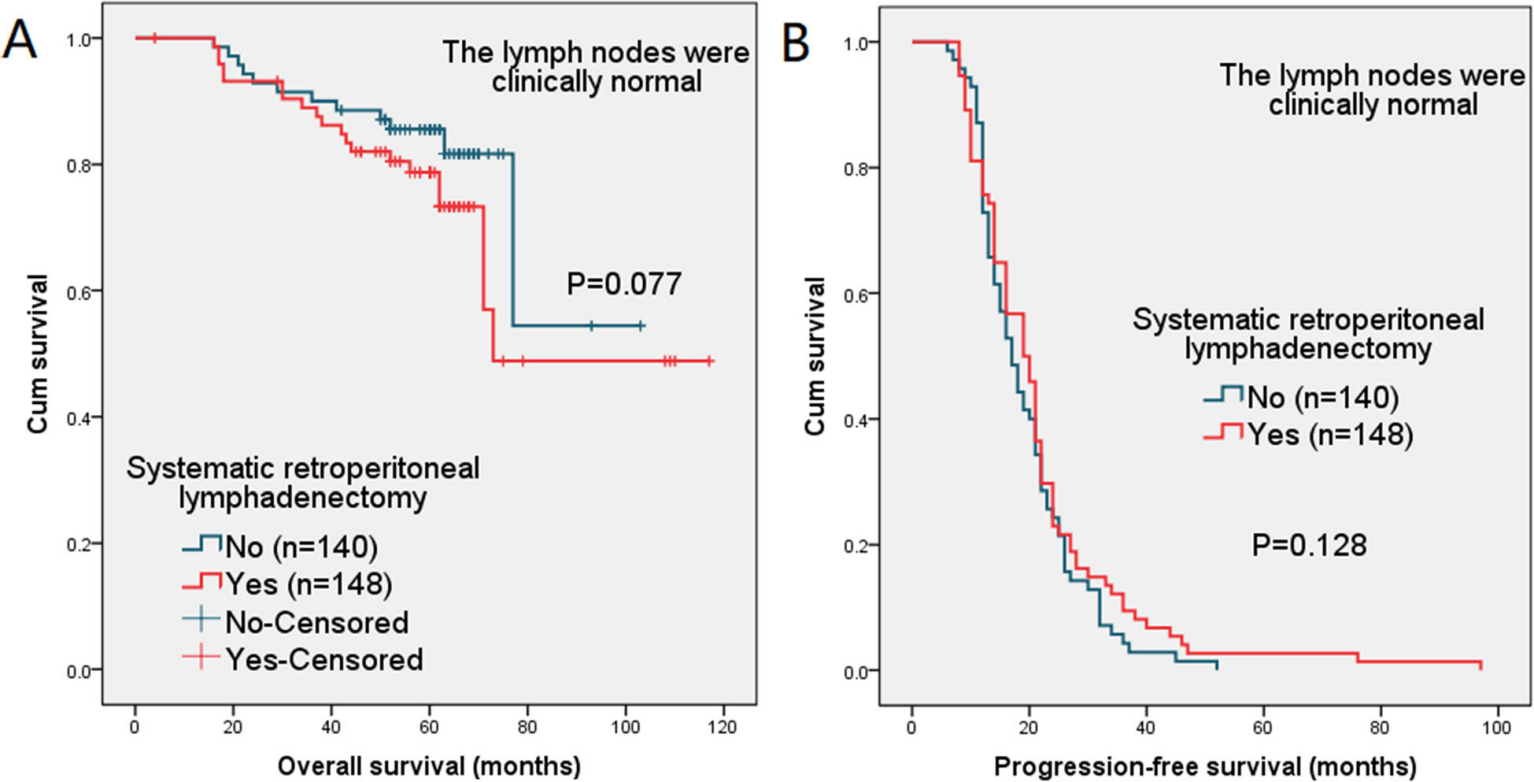
(**a**) Overall survival (OS) and (**b**) progression-free survival (PFS) in patients with negative lymph nodes

In subgroup analysis of patients with negative lymph nodes (including evaluation of preoperative imaging and intraoperative exploration), the difference in the 5-year OS and 2-year PFS was also not statistically significant (*P* = 0.077 and 0.128, respectively). The survival curves are shown in Fig. 4.

#### FIGO stage IIIB-IV

**Fig. 5:**
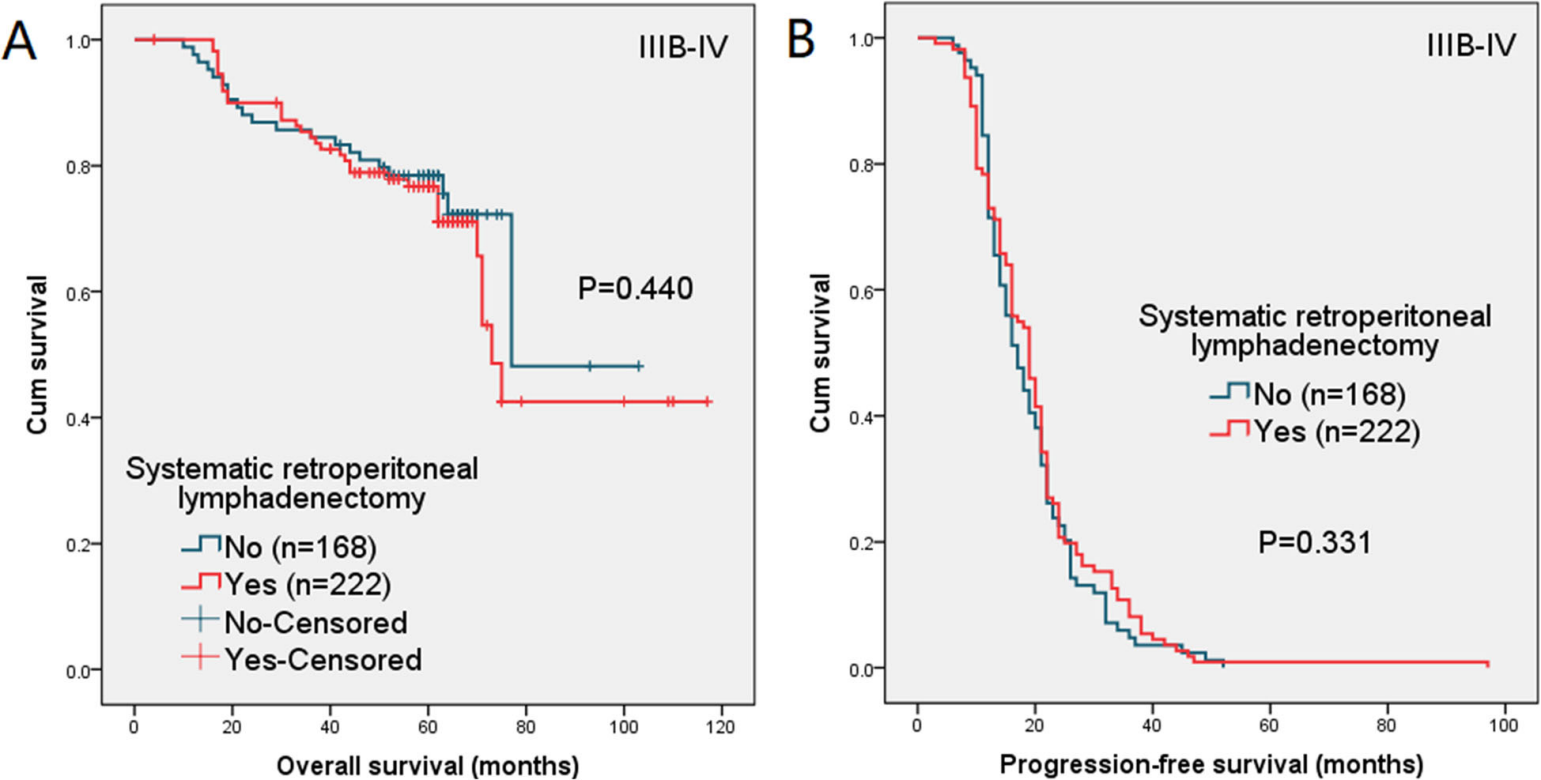
(**a**) Overall survival (OS) and (**b**) progression-free survival (PFS) in patients with FIGO stage IIIB-IV

In the separate analysis of patients with macroscopic peritoneal metastasis beyond pelvic (FIGO stage IIIB-IV), there was no significant difference in 5-year OS and 2-year PFS between the two groups (*P* = 0.440 and 0.331, respectively). The survival curves are presented in Fig. 5.

### Multivariate analysis of clinicopathologic factors in relation to PFS and OS of patients (Table 3)

A multivariate Cox regression model was established in this study, FIGO stage (III/IV), histological types (serous/non-serous), and lymphadenectomy (no/yes) were imported into this model. The results showed that, systematic retroperitoneal lymphadenectomy was not a significant factor influencing the patients’ survival.

**Table 3:** Multivariate analysis of clinicopathologic factors in relation to PFS and OS of patients

	No	Progression-free survival (PFS)		Overall survival (OS)	
		Hazard ratio (95% CI)	P-value	Hazard ratio (95% CI)	*P*-value
Total	410				
FIGO stage					
III	342	1	0.006	1	0.403
IV	68	0.694 (0.534–0.902)		0.804 (0.483–1.340)	
Histology					
Serous type	320	1	0.521	1	0.725
Non-serous type	90	0.926 (0.731–1.172)		1.082 (0.697–1.680)	
Lymphadenectomy					
No	170	1	0.237	1	0.389
Yes	240	1.127 (0.924–1.374)		0.846 (0.578–1.238)	

## Discussion

Lymph node metastasis is one of the main metastatic pathways of ovarian cancer, with a total probability of 20 to 41%, while retroperitoneal lymph node metastasis rate of advanced ovarian cancer is as high as 50 to 75% [12, 13]. There are three main ways to remove lymph nodes: lymph node sampling, removal of palpable nodes and systematic/radical lymphadenectomy. Systemic retroperitoneal lymphadenectomy refers to the complete removal of lymphatic and adipose tissue around the abdominal aorta and inferior vena cava, as well as the pelvic cavity on both sides, generally last to the level of the left renal vein, the lower boundary to the inguinal ligament level. And bilateral psoas, anterior longitudinal ligament of the spine and sacral periosteum should be exposed and visible after surgery [14].

Some studies indicated survival benefit of lymphadenectomy in patients with early-stage ovarian cancer. Chan JK et al. [15] conducted a retrospective study on 6686 patients with stage I ovarian cancer in 2007, and showed that lymphadenectomy improved the 5-year survival rate of epithelial ovarian cancer patients with non-clear cell carcinoma.

However, results of studies on whether systemic retroperitoneal lymphadenectomy can improve the prognosis of advanced ovarian cancer patients were different. The majority of early retrospective studies have suggested a favorable prognosis of systematic retroperitoneal lymphadenectomy in patients with macroscopically completely resected advanced ovarian cancer. du Bois A et al. [6] reviewed 1942 epithelial ovarian cancer patients, the results showed that among the 996 patients without residual tumor, the 5-year survival rate was significantly higher in the group receiving lymph node resection of different degrees than that in the group without lymph node resection (67.4% vs 59.2%, *P* = 0 .0166); besides, lymphadenectomy showed a significant survival influence on those patients without clinically suspected nodes (the median OS was 108 vs 83 months, *P* = 0.0081); meanwhile, patients with small residual tumor also showed a positive effect on lymphadenectomy regardless of clinical lymph node status. A retrospective study consisting of 488 patients with untreated advanced ovarian cancer also revealed that among patients with optimal or suboptimal cytoreduction, 5-year survival in patients who underwent lymphadenectomy was higher than the patients who did not (*P* = 0.05, *P* < 0.005) [7]. Aletti GD et al. [8] also demonstrated a favorable prognosis in the stage IIIC/IV epithelial ovarian cancer patients who received lymphadenectomy, in which 5-year OS was 50% (lymphadenectomy) vs 33% (lymph node sampling) vs 29% (no lymph node assessment) (*P* = 0.01). Chan JK et al. [9] reported that among stage III-IV ovarian cancer patients, expanding the scope of lymph node resection can improve the survival rate. A comparative study on patients with advanced ovarian cancer (stage IIIC-IV) and no residual disease showed that systematic pelvic and para-aortic lymphadenectomy significantly improved patients’ survival (*P* = 0.02) [10]. Burghardt et al. [16] analyzed stage III ovarian cancer patients, also found a superior prognosis of lymphadenectomy. Kikkawa et al. [17] indicated that the incidence of death in the lymphadenectomy group was lower than that in the control group (Hazard Ratio: 0. 677; P = 0. 0497).

However, a number of studies have reported that systematic pelvic and para-aortic lymphadenectomy has no benefit to patients’ prognosis.

Spirtos NM et al. [18] reviewed the role of retroperitoneal lymphadenectomy in patients with stage IIIA-IVA advanced ovarian cancer who underwent suboptimal cytoreductive surgery (residual tumor was < 1 cm), the result uncovered that patients who underwent removal of macroscopically positive lymph nodes had no superiority in terms of benefits than those with microscopically positive and/or negative lymph nodes. Sakai K et al. [3] also reported among the advanced ovarian cancer patients with optimal cytoreduction (residual tumor < 1 cm), there was no significant difference in 5-year OS (59 vs 62.9%, *P* = 0.853) or PFS (41.9 vs 46.7%, *P* = 0.658) between patients who underwent systematic retroperitoneal lymphadenectomy and others. In addition, there was no therapeutic benefit for advanced ovarian cancer patients who underwent systematic retroperitoneal lymphadenectomy during interval debulking surgery after neoadjuvant chemotherapy [19].

Based on the results achieved in our study, no remarkable improvement was noted in survival of advanced ovarian cancer patients with optimal or suboptimal cytoreduction who underwent systematic retroperitoneal lymphadenectomy (either 2-year PFS or 5-year OS).

Panici PB et al. [12] conducted a randomized clinical trial in 2005, and randomly divided 427 patients with optimally debulked advanced ovarian cancer (stage IIIB-IV) to systematic pelvic and para-aortic lymphadenectomy group (*n* = 216) and resection of bulky nodes only group (*n* = 211). After a median follow-up of 68.4 months, the risk of recurrence was significantly lower in the systematic lymphadenectomy group (hazard ratio [HR] = 0.75, 95% confidence interval [CI] = 0.59–0.94; *P* = 0.01) than in the no-lymphadenectomy group, while the risk of death was similar in both groups (HR = 0.97, 95% CI = 0.74–1.29; *P* = 0.85). The majority of ovarian cancer patients treated in our hospitals had macroscopic peritoneal metastasis beyond pelvic. Thus, in the current research, we also performed a subgroup analysis of stage IIIB-IV ovarian cancer patients. Our findings indicated that lymphadenectomy had no significant effect on patients’ survival, 5-year OS rate was 77 and 78% in the lymphadenectomy group and no-lymphadenectomy group, *P* = 0.440; 2-year PFS was 26 and 24% in the two groups, *P* = 0.331.

Patients with serous ovarian cancer has a higher rate of lymph node metastasis than other types of epithelial ovarian tumors [20]. Takeshima N et al. [21] carried out an analysis of 208 ovarian cancer patients with systematic lymphadenectomy: 60 cases of serous tumor, 22 had positive lymph nodes (36.7%); 148 cases of Non-serous tumor, 25 had positive lymph nodes (16.9%). In this study, patients with serous tumor and non-serous tumor were analyzed separately. As the data showed, no matter whether the tumor was serous type or not, systematic retroperitoneal lymphadenectomy was not a prognostic factor for PFS or OS.

Lymphadenectomy in patients without clinically suspect lymph nodes and small residual disease intraperitoneally might not change the residual disease status but may reduce tumor burden that is possibly resistant to chemotherapy. In the Lymphadenectomy in Ovarian Neoplasms (LION) trial, 647 patients with newly diagnosed advanced ovarian cancer (FIGO stage IIB-IV) who had undergone macroscopically complete resection and had normal lymph nodes both before and during surgery were intraoperatively randomly assigned to lymphadenectomy and no lymphadenectomy groups. It was revealed that systematic pelvic and paraaortic lymphadenectomy in these patients was not associated with longer survival than no lymphadenectomy and was associated with a higher incidence of postoperative complications, such as incidence of lymph cysts, infection treated with antibiotics, repeat laparotomy and mortality within 60 days after surgery [11]. Similarly, in the present study, a subgroup analysis of the patients with clinically negative lymph nodes, showed that there was also no survival benefit for patients who underwent systematic lymphadenectomy.

## Conclusions

Routine systematic pelvic and paraaortic lymphadenectomy does not confer any survival benefit in advanced ovarian cancer patients who have no gross residual disease or residual disease < 1 cm at the end of resection, while unnecessary surgical procedure increases the risk of postoperative complications (e.g., lymph cysts, etc.). This was a retrospective study conducted at a single institution; thus, the limitation of data collection was tangible.

## Data Availability

The datasets used and analyzed during the current study are available from the corresponding author on reasonable request.

## Abbreviations

FIGO: Federation of Gynecology and Obstetrics
PFS: Progression-free survival
OS: Overall survival
HR: Hazard ratio
N/A: Not applicable
